# Gender-dependent color discrimination ability and speed–accuracy tradeoff: insights from ND-100 hue tests

**DOI:** 10.3389/fpsyg.2025.1722974

**Published:** 2026-01-05

**Authors:** Yanan Qiao, Yasuhiro Kawabata, Mikuko Sasaki

**Affiliations:** 1 Graduate School of Humanities and Human Sciences, Hokkaido University, Sapporo, Hokkaido, Japan; 2 Japan Color Research Institute, Saitama, Japan

**Keywords:** ND-100 hue test, color association, color discrimination, time limit, vision, speed-accuracy tradeoff

## Abstract

**Background:**

Time pressure influences perceptual decisions, but its effects on speed—accuracy tradeoff (SAT) and decision processes in color discrimination remains poorly understood, particularly regarding potential gender differences.

**Objective:**

To systematically examine how varying time pressures influence color discrimination performance, SATs, and underlying decision processes in males and females.

**Methods:**

A total of 356 university students (18–28 years) completed the ND-100 hue test under four time conditions (120, 105, 90, and 75 s). Each participant performed up to four trials to reduce task unfamiliarity effects. The study introduced three methodological innovations: (1) the first use of a 75-s time limit to model high-pressure conditions; (2) a fine-grained range of time limits (75–120 s) to capture detailed performance changes; and (3) multiple trials per participant to minimize learning and unfamiliarity effects.

**Results:**

Under moderate time limits (90–120 s), females outperformed males by 19.889–29.926 points in total error scores. At the most stringent time limit (75 s), no significant gender difference was observed (*p* = 0.918), indicating convergence of performance under extreme time pressure. Both sexes exhibited a clear SAT, with females’ performance declining more sharply at 75 s, suggesting differential reliance on analytical processing, experience-based strategies, and decision-threshold adjustments.

**Mechanistic interpretation:**

The observed effects are interpreted within an integrative triple-mechanism framework, in which time pressure modulates the dynamic interplay among: (1) biological predispositions (e.g., photopigment variation, P-cell density differences, and interhemispheric connectivity differences); (2) experience-dependent perceptual plasticity; and (3) adaptive cognitive strategies for decision making.

**Conclusions:**

This study provides the first systematic evidence of gender differences in color discrimination performance under extreme temporal constraints, supports an integrative biological—experiential—cognitive model of time-pressured perception, and offers practical implications for training, task design, and interface development in real-world contexts requiring rapid color-based decisions.

## 1 Introduction

Beyond serving as a perceptual attribute of objects, color is a fundamental dimension of visual neural processing. Color perception arises from the absorption of light by cone photoreceptors and subsequent processing along the visual pathway (Gegenfurtner and Kiper, 2003; Conway, 2009). Through these hierarchical transformations, color information contributes to the categorization and recognition of objects, via multiple aspects, including hue, saturation, lightness, and the spatial distribution of colors, which together provide distinctive cues for differentiating object classes (Ge et al., 2022). Rather than being a mere surface property, color functions as a key cue that organizes visual input and interacts with attention, memory and emotions (Bannert and Bartels, 2018; Conway, 2009). Understanding how the visual system represents and differentiates chromatic signals is therefore crucial not only for perceptual research but also for elucidating the neural mechanisms that support complex visual behaviors relevant to safety, health, and communication.

### 1.1 Visual physiology

Color perception begins with the absorption of light by cone photoreceptors in the retina, representing the initial stage of neural encoding (Gegenfurtner and Kiper, 2003; Conway, 2009). Signals from the L-, M-, and S-cones are transmitted to retinal ganglion cells and relayed via the multilayered lateral geniculate nucleus of the thalamus to primary visual cortex (V1), where chromatic and achromatic information are processed in opponent channels and integrated into higher-order representations, including hue, saturation, lightness, and color pattern information (Gegenfurtner and Kiper, 2003). In V1 and higher visual areas, these signals undergo hierarchical processing, enabling the integration of chromatic information with texture and contextual features of the visual scene (Solomon and Lennie, 2007). Electroencephalography (EEG) evidence further shows that color-specific neural responses differ across cortical regions: Khadir et al. (2023) found that green stimuli elicited earlier β-band responses in the occipital cortex compared with red and blue, highlighting color-specific neural dynamics in early sensory processing.

Individual differences in color perception arise not only from variations in cone photopigments but also from higher-level neural processing, including cortical visual areas (V2 and V4) and cognitive mechanisms such as memory and color categorization. Genetic polymorphisms influencing cone opsins can alter spectral sensitivity (Dees et al., 2015), while aging and variations in lens or macular pigment can influence the light spectrum reaching the retina (Opoku-Yamoah et al., 2025). Furthermore, the human color perception system may adjust its sensitivity to align with the statistical regularities of colors encountered in the environment, reflecting effects of prolonged experience or exposure (Skelton et al., 2021). Evidence from animal studies indicates that higher visual cortical areas exhibit experience-dependent plasticity, showing distinct functional adaptations in response to altered sensory input (Craddock et al., 2023), which supports the notion that human color vision may similarly be shaped by long-term environmental exposure. fMRI studies show that color-selective responses are present in V1 and V2 and are increasingly enhanced in anterior regions such as V4 and VO in macaque, reflecting hierarchical processing of chromatic information (Lafer-Sousa and Conway, 2013). Beyond sensory encoding, color-based decision-making engages higher-order neural circuits that dynamically modulate perceptual strategies under time constraints; for example, the caudate nucleus adjusts decision thresholds, with higher activity linked to slower, more accurate responses and lower activity linked to faster, potentially less accurate decisions (Yau et al., 2020). These findings indicate that individual differences in color discrimination and speed–accuracy tradeoffs (SATs) may arise from interactions between sensory hierarchies and subcortical circuits regulating response strategies, providing a neurobiological framework for understanding gender-dependent performance under varying time pressures. Overall, color vision emerges from a multilevel interplay of retinal, thalamic, and cortical mechanisms, forming a foundation for studying both the sensory encoding and cognitive modulation of color perception.

### 1.2 Factors influencing color discrimination performance

Extensive research has investigated the factors that contribute to individual differences in color vision. From a physiological perspective, Elliot et al. (2015) conducted a color-matching study in which participants adjusted three spectral lights, red, green, and blue, to reproduce the presented hues. Individuals with normal color vision (trichromats) successfully combined all three lights to match any color. By contrast, dichromats, who can distinguish only two primary color channels, relied on two lights, while participants with achromatopsia required only one light to match any hue (Elliot et al., 2015). Protanopia in dichromacy results from loss of the long-wavelength sensitive mechanism in the retina, and deuteranopia lacks the normal green-sensitive pigment (Fletcher and Voke, 1985). These results underscore the role of physiological differences in shaping color vision performance and perception (Emery and Webster, 2019).

Aging also leads to changes in color vision. After the age of 30, red–green thresholds increase linearly at a rate of 1% per year and blue–yellow thresholds increase at a rate of 1.6% per year (Opoku-Yamoah et al., 2025).

Pathological conditions also affect color vision. Kim et al. (2024) demonstrated that patients with Parkinson’s disease and isolated rapid eye movement sleep behavior disorder (iRBD) exhibited reduced color discrimination compared with healthy individuals. Similar impairments have been reported in patients with depression (Deisenhammer et al., 2022), schizophrenia (Dahdouh et al., 2022), and myopia (Panchal et al., 2012).

Sociodemographic factors, such as sex, also influence color discrimination. Early work by Verriest et al. (1962) suggested sex-based differences, which more recent findings have consistently supported. For instance, Imbery et al. (2018) used the Farnsworth–Munsell 100 hue test with a sample of 95 first-year dental students from diverse ethnic backgrounds (Caucasian, Black, Asian, and Hispanic). They found that females performed considerably better than males. Similarly, Panchal et al. (2012) tested 100 Indian medical students with the same method and reported better performance among females. These findings were further replicated by Gupta et al. (2020) in a study of 170 young adults, confirming that females consistently outperform males in color discrimination tasks. Bimler and Bonnardel (2018) found that females exhibited more consistent and fine-grained perceptual color scaling than males, suggesting that female observers may possess slightly superior color discrimination organization.

Professional and experiential factors, such as participation in vocational courses, club activities, or occupations involving color-related tasks, can also influence color discrimination ability. A previous study showed that trichromats with more than 3 years of experience in art clubs or color-focused classes performed better in discrimination tasks than those with no experience or less than 3 years of exposure (Kawabata et al., 2020). Similarly, Matsumoto et al. (2021) reported that workers in the color-related furniture industry exhibited superior color discrimination. Lovell and Rabin (2021) further demonstrated that jewelry appraisers had considerably lower total error scores (TESs) in color discrimination, within the expected range for their age group. In addition to long-term exposure, short-term training also has an impact on performance. Horiuchi and Nagai (2024) conducted a 5-day program comprising five training sessions, each consisting of 200–300 trials. They found that exposure to colors along the positive and negative directions of the L–M axis reduces discrimination thresholds and alters the perception of those colors.

From a cross-cultural perspective, variations in race and lifestyle have also been shown to affect color perception. For example, Mantyjarvi (2001), using the Farnsworth–Munsell 100 Hue Test in participants aged 30–49 years from different nationalities, found that individuals from the Democratic Republic of the Congo have poorer discrimination compared with those from Finland. Lifestyle factors also play a role. The TES on the Farnsworth–Munsell 100 Hue Test for smokers was positively correlated with the number of cigarettes smoked per day, suggesting that higher tobacco use impairs discrimination (García-Domene et al., 2022). Likewise, no-alcohol drinkers exhibit better color discrimination compared with those who drink (Brasil et al., 2015).

Beyond individual differences in color discrimination, imposing a time constraint on the task considerably influences performance. In a seminal study, Yellott (1971) applied eight time-limit conditions (150–800 ms) in a red–green discrimination experiment and found a strong positive correlation between available time and response accuracy, which increased from 0.74 to 0.99. More recently, Kawabata et al. (2020) demonstrated that error rates in color discrimination decrease when participants are given longer completion time (120 vs. 90 s). Together, these findings highlight a speed–accuracy tradeoff (SAT) in color discrimination: performance accuracy improves as the allotted time increases.

Despite its importance, research on gender differences in color discrimination under time constraints remains limited. Existing studies often suffer from methodological shortcomings that limit their generalizability. Several previous studies exhibited specific methodological limitations that constrained their broader applicability. For instance, Yellott (1971) examined a wide range of time constraints but focused exclusively on red–green discrimination, limiting applicability to other hues. Imbery et al. (2018) included ethnically diverse participants but imposed no time limits and used small, unequal group sizes, which may have reduced statistical power and introduced sampling bias. Gupta et al. (2020) provided participants with an extended 2.5-min response window but employed an unbalanced gender ratio, potentially biasing observed gender differences. Kawabata et al. (2020) used ND-100 hue test and compared 2-time conditions (120 and 90 s), leaving intermediate performance changes undetected. Additionally, many studies using the Farnsworth–Munsell 100 Hue Test relied on a single trial per participant, inflating error scores (ESs) due to task unfamiliarity and potentially misclassifying normal trichromatic observers. Rodríguez-Carmona et al. (2008) reported that females had higher thresholds in Color Assessment and Diagnosis test (CAD) than males along the red–green (RG) axis but no difference along the yellow–blue (YB) axis. However, the study did not examine task complexity, condition variations, other hues, or continuous color gradients. Jaint et al. (2010) found higher female accuracy in RG recognition but used simple matching tasks without time limits. Mcgivern et al. (2019) also found females more accurate in color judgment, yet tasks lacked time pressure and did not assess continuous color gradients. These methodological limitations reduce the reliability and generalizability of prior findings and hinder a systematic understanding of fine-grained color discrimination, limiting the applicability of results to more dynamic or real-world contexts. Importantly, these behavioral findings suggest that individual and situational factors modulate color discrimination not only at the perceptual level but also through neural mechanisms. EEG studies show color-specific responses in occipital regions, with green stimuli eliciting earlier β-band activity than red or blue (Khadir et al., 2023). Functional magnetic resonance imaging (fMRI) evidence demonstrates experience-dependent plasticity in higher visual cortical areas, reflecting functional adaptation to sensory input (Craddock et al., 2023). Moreover, the anterior cingulate cortex adjusts decision-threshold distance based on available predictive information, while the dorsolateral prefrontal cortex encodes sensory evidence accumulation (Domenech and Dreher, 2010). Together, these neural mechanisms integrate biological predispositions, experience-dependent plasticity, and cognitive strategies, shaping performance under time pressure and contributing to observed speed–accuracy tradeoffs.

### 1.3 Present research

Based on the limitations of previous research on gender differences in color discrimination under time constraints (as discussed in section 1.2), the present study addresses these limitations by investigating gender differences in an Asian sample using shorter time limits to better approximate real-world conditions. Specifically, we examined whether time pressure moderates gender-related differences in color discrimination performance. To ensure validity and representativeness, we employ a large, gender-balanced sample. The experiment utilizes the ND-100 hue test (Japan Color Enterprise Co., Ltd., Tokyo, Japan). Additional technical details are provided by Nippon Color & Design Research Institute Inc. (2025), which is derived from the well-known Farnsworth–Munsell 100 Hue Test (Farnsworth, 1943) frequently cited in previous research. The two versions differ slightly in color composition and the number of test items. The ND-100 hue test represents 10 hues, red, orange, yellow, yellowish-green, green, bluish-green, blue, bluish-purple, purple, and reddish-purple, arranged on a circular scale. Although initially designed to detect color vision deficiencies, the test has proven effective in evaluating color discrimination in individuals with normal trichromatic vision by using error scores as a performance index.

To improve the investigation of time-constraint effects, we introduce two additional time limits, 105 and 75 s, in addition to those used in previous studies (120 and 90 s) (Kawabata et al., 2020). The 105 s condition serves as an intermediate point to determine whether performance declines linearly or exhibits non-linear variation. The novel 75 s condition simulates a highly time-pressured environment, enabling us to examine how individuals their color discrimination strategies under extreme constraints and whether their perceptual advantage persists. Incorporating these new conditions provides a strong empirical basis for understanding the dynamic effects of time pressure on color discrimination and gender differences.

Furthermore, we include between one and four trials per participant and applied a weighted integration method to balance the average order of trial conditions. This procedure minimizes practice-related bias, enhances comparability across time limits, and ensures a more robust and reliable assessment of color discrimination.

Accordingly, the study investigates the moderating effect of time pressure on gender differences in color discrimination by comparing the performance of males and females under progressively shorter time limits. We propose the following hypotheses:

*H1*: Female participants are expected to show better color discrimination performance than male participants under all shortened time-limit conditions (75, 90, and 105 s), with the largest gender difference anticipated under the most extreme time constraint (75 s).

*H2*: Color discrimination performance across the full hue circle is expected to decrease progressively as time limits are reduced (120 s → 105 s → 90 s → 75 s). As females generally demonstrate higher accuracy in color discrimination tasks, the decline in performance is expected to be greater in males, particularly under the most demanding time constraint (75 s).

This study advances research on color discrimination under time pressure by introducing a more finely graded set of time limits: 120, 105, 90, and 75 s. Previous studies used broader intervals, which limited the understanding of dynamic changes in discrimination accuracy. By incorporating both intermediate and extreme short-time conditions, this study offers higher-resolution insights into how time constraints affect performance. It also systematically examines gender differences across these conditions, exploring whether females maintain their detail-processing advantage under extreme pressure. To improve reliability, the study used a large, gender-balanced sample and repeated measures, minimizing the impact of unfamiliarity during early trials. The integration of multilevel time constraints into a 100-hue task enhances ecological validity and experimental precision. Together, these innovations contribute to visual cognition and gender research by revealing how time pressure influences perceptual strategies, and they provide practical implications for improving cognitive performance in fast-paced environments.

### 1.4 Innovation

This study introduces three methodological innovations that advance current understanding of factors influencing color discrimination. First, it is the first to employ a 75 s time limit to model high time pressure conditions. Second, it employs a fine-grained set of time limits (120, 105, 90, and 75 s), allowing detailed observation of changes in discrimination performance. Third, it incorporates up to four trials per participant, reducing inflated error due to initial unfamiliarity and thereby improving measurement stability and reliability. Together, these enhancements improve the precision and ecological validity of time-constrained color discrimination research, thereby deepening insight into gender-related differences. This approach also advances research on visual cognition by clarifying how time pressure shapes perceptual strategies, with practical implications for optimizing cognitive performance in fast-paced environments.

These methodological innovations are valuable because they increase the precision and ecological validity of color discrimination research under time pressure. The use of a 75-s limit offers a more pragmatic model of high-pressure conditions, while the fine-grained time intervals (120, 105, 90, and 75 s) allow the detection of incremental fluctuations in performance. Incorporating multiple trials per participant mitigates errors due to task unfamiliarity, thereby enhancing measurement reliability. Collectively, these enhancements not only strengthen the validity of findings on gender differences in color discrimination but also advance visual cognition research by clarifying how time pressure modulates perceptual strategies. Practically, they offer insights applicable to domains requiring rapid visual decision-making. This study is the first to systematically investigate the effect of gender differences on color discrimination under extremely short time limits, addressing a key gap in understanding how time pressure shapes perceptual performance.

## 2 Materials and methods

### 2.1 Participants

This study was reviewed and approved by Center for Experimental Research in Social Sciences (CERSS), Hokkaido University and conducted in accordance with the ethical standards of the Declaration of Helsinki (1996). Written informed consent was obtained from all participants.

A total of 356 Asian college students, aged 18–28 years (mean age = 19.428 ± 1.747), participated in the study. The sample comprised 179 males and 177 females. Participants were recruited from psychology courses and lectures, and they received extra course credit for their participation. One participant over the age of 30 was excluded to maintain the intended age range of 18–28 years and avoid potential age-related bias. In addition, two participants who demonstrated abnormal color vision during screening were removed from the dataset. All remaining participants had normal or corrected-to-normal visual acuity and normal color discrimination. Color discrimination ability was assessed using the ND-100 hue test. Each participant completed four test trials, and data were collected from all four trials. Although the number of participants completing each repetition varied across time-limit conditions, weighted averages were calculated for each condition to standardize trial sequences across groups. This approach improved comparability between conditions by minimizing potential confounding effects of unequal trial repetitions and reducing bias from inexperience in the initial trial. To further address potential inexperience in the first trial, all four trials were included in the analysis, and weighted averages were calculated for each condition.

Notably, the sample consisted only of Asian university students aged 18–28 years. This restriction may limit the generalizability of the findings to other age groups, cultural backgrounds, or populations with different educational experiences. Cultural differences in color perception or experience may influence performance (e.g., Bortolotti et al., 2023; Singh and Srivastava, 2011; Bortolotti et al., 2025). Therefore, the present results should be interpreted with caution when extrapolating beyond the studied population.

Power analysis was conducted using G*Power 3.1 (Faul et al., 2007), developed at Kiel University, Germany. An *a priori* power analysis using G*Power 3.1 (Faul et al., 2007) was performed for a repeated-measures ANOVA with a within–between interaction. The analysis assumed α = 0.05, desired power = 0.95, two groups (male vs. female), four repeated measurements (time-limit conditions: 120, 105, 90, and 75 s), a correlation among repeated measures of 0.5, and a non-sphericity correction (ε) of 1. Based on a partial η^2^ = 0.333 (effect size *f* = 0.707), the required total sample size was estimated to be eight participants, with an actual achieved power of 0.994. The number of participants in this study far exceeded the minimum requirement, ensuring robust statistical power to detect gender differences under varying time constraints. The average trial order and the total number of trials for each condition are summarized in Table 1.

**TABLE 1:** Average order and number of trials.

Time limits (s)	Order of trials	Number of trials
120	2.776 (± 1.479)	152
105	2.171 (± 0.948)	234
90	2.115 (± 0.834)	227
75	2.793 (± 0.880)	208

### 2.2 Stimuli and apparatus

Although color vision is a subjective and emergent perceptual experience, standardized tests enable its objective quantification by assessing distinct components (Lakowski, 1969a). These tests can be administered relatively quickly (Lakowski, 1969b). In this study, we employed the ND-100 hue test.

The ND-100 hue test consists of a large number of color samples and enables detailed assessment of hue discrimination (Rigby et al., 1991). It is commonly used in research and industry to evaluate color perception (Yang et al., 2005; Gupta et al., 2020). The test is based on the well-established Munsell 100-Hue Test (Farnsworth–Munsell), which has been widely validated in the previous study (Kawabata et al., 2020). Test–retest reliability of the Munsell 100-Hue Test typically falls within the moderate-to-high range (*r* ≈ 0.7–0.8), indicating stable performance across repeated administrations (Anderson and Johnston, 2014). Reliable individual differences have also been reported among observers with normal color vision, suggesting that the test can capture meaningful variation in chromatic discrimination ability. Given its equivalent design and calibration procedures, the ND-100 hue test can therefore be considered a reliable and standardized tool for assessing fine-grained hue discrimination.

**FIGURE 1:**
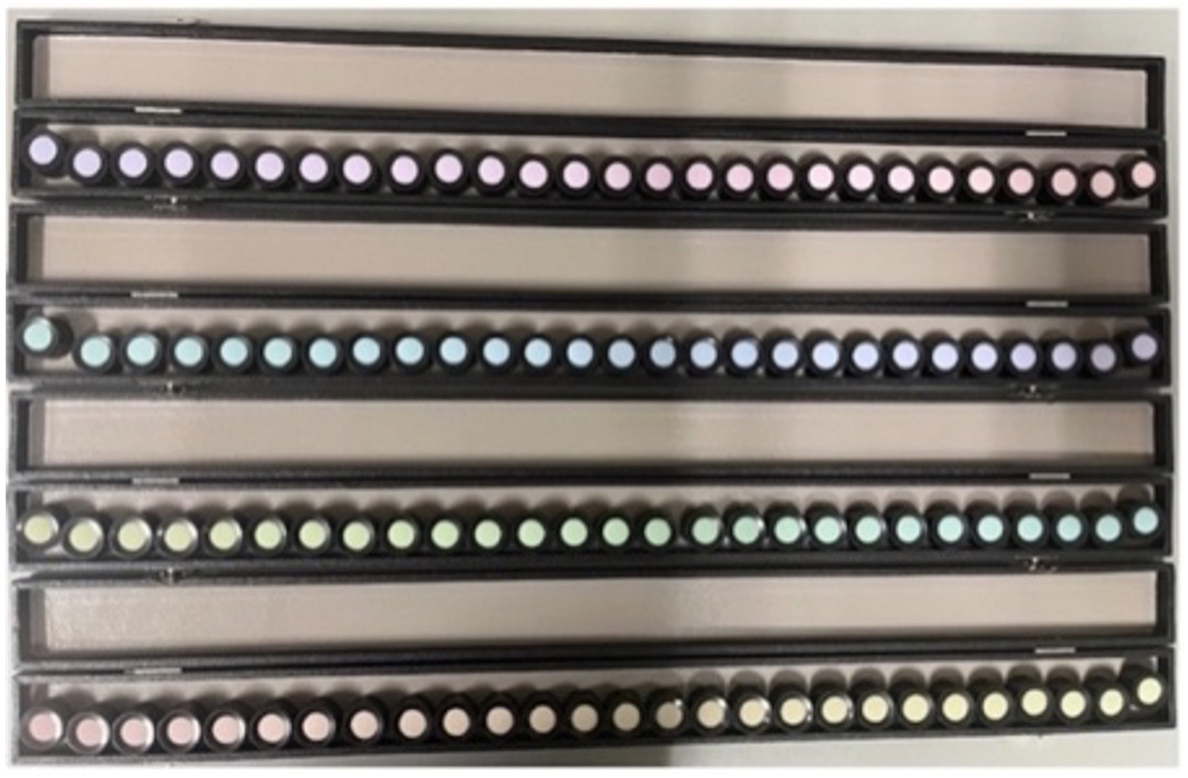
Experimental apparatus. The test contains 100 colored caps, spanning from red to reddish-purple; From bottom to top, Box 1 contains red to yellowish-green caps, Box 2 contains yellowish-green to bluish-green caps, Box 3 contains from bluish-green to bluish-purple caps, and Box 4 contains bluish-purple to red caps. Each box includes two fixed reference caps at both ends. The caps are designed with approximately uniform lightness (Munsell value ≈ 6) and form a continuous hue gradient when arranged correctly.

The apparatus used for the ND-100 hue test is shown in Figure 1. It consists of 4 boxes, each containing 25 movable color caps that served as experimental stimuli. Two fixed caps were placed at both ends of each box as reference anchors, helping participants arrange the movable caps in a perceptually continuous hue sequence. Each movable cap is numbered on the reverse side, corresponding to its original position, which enables calculation of the participant’s error score (ES). The ES represents the degree of mismatch between each cap and its neighbors (Kawabata et al., 2020). When caps are correctly arranged, the absolute difference between adjacent numbers equals 1. Misplaced caps result in larger differences, producing higher ES values (an ES of 0 indicating that all caps and their neighbors are correctly ordered) (Kawabata and Kawabata, 2014). When the caps are perfectly ordered, the ES equals 0, producing a smooth and continuous hue gradient across all boxes. The sum of the ES for all 100 color caps is referred to as the total ES (TES). To evaluate the reliability of TES scoring across repeated trials, an intraclass correlation coefficient (ICC) analysis was conducted using a two-way mixed-effects model for consistency [ICC(C,4), average measures]. The results demonstrated excellent internal consistency, ICC = 0.936, 95% CI [0.899, 0.962], *F* (44,132) = 15.6, *p* < 0.001, indicating minimal within-subject measurement error across repeated trials. Between-subject variability was also substantial, as shown by the range of TES scores across participants, supporting the method’s sensitivity for detecting individual differences in color discrimination performance. Overall, these findings suggest that the TES scoring method is reliable and capable of capturing meaningful variability between participants, thereby minimizing concerns about measurement error influencing the study’s conclusions.

All test caps were visually inspected prior to testing to ensure there was no discoloration or surface damage. The order of test caps was randomized for each trial to prevent sequence effects. The light source in all boxes was a dimmable D65 lamp (Z-209PROB, Yamada Shomei Co., Ltd., Tokyo, Japan), which served as the standard daylight illuminant (Bustamante et al., 2021). During the experiment, illuminance levels were maintained between 100 and 2,000 lux, consistent with standard procedures for the Farnsworth–Munsell 100-Hue Test (Boyce and Simons, 1977). The illuminance levels applied in our experiments are summarized in Table 2. Before each experimental session, all lighting equipment was inspected to confirm it was undamaged. To maintain consistent illumination across sessions, curtains were drawn to reduce stray light, and participants were instructed to set the lamp to levels 3 or 4 throughout all sessions, ensuring that illuminance remained within the desired range.

**TABLE 2:** Experimental illuminance and coordinates in the CIE 1931 color space.

		CIE1931*	
Time limits (s)	Average illuminance (lux)	X	Y
120	1,190 (± 461)	0.3134 (± 0.0025)	0.3268 (± 0.0034)
105	1,209 (± 520)	0.3140 (± 0.0096)	0.3267 (± 0.0107)
90	1,087 (± 556)	0.3131 (± 0.0112)	0.3270 (± 0.0097)
75	1,222 (± 495)	0.3135 (± 0.0098)	0.3262 (± 0.0102)

*The CIE 1931 color space models human color perception using Yxy coordinates, where Y represents luminance.

### 2.3 Procedure

Participants were tested in groups of four, with group members randomly assigned. Before beginning, they were given a 15 min adaptation period to acclimate to the experimental illumination conditions. During this period, the experimenter explained the study’s purpose and provided instructions on operating the apparatus, which consisted of four boxes, each containing 27 color caps serving as stimuli. The two end caps in each box were fixed, while the remaining 25 could be freely rearranged during the allotted time. At the start of each trial, participants were to reorder the 25 movable caps randomly. They were then asked to rearrange them between the two fixed reference caps so that the sequence formed a smooth and continuous transition of colors.

Before each trial, participants received standardized verbal and written instructions outlining the task requirements, the procedure for arranging the color caps, and a demonstration of correct sorting. They were instructed to maintain focus throughout each trial, and any questions were addressed before the experiment began. Instructions on time limits were also provided. During the task, the experimenter gave verbal prompts every 30 s to inform participants of the remaining time and assist with pacing. Participants were tested in groups of four in the same room. To minimize potential social or familiarity effects, friends were not placed in the same group, and each group was composed of approximately two males and two females. Each participant sat at an individual table with adequate spacing and focused on the color caps under standardized illumination. Although group testing may introduce subtle social or competitive influences, the experimental design and setup were carefully controlled to minimize such effects. Nonetheless, the potential impact of group testing should be acknowledged as a methodological consideration, and future studies may conduct individual testing to fully eliminate social influences. This experiment investigated color identification under time pressure by introducing refined time constraints of 120, 105, 90, and 75 s. Participants completed the ND-100 hue test under all four time constraints, arranging the 25 color caps in each box within the allotted time. Under each condition, all four boxes of the 100-hue test were completed once, with participants arranging the 25 color caps in each box within the allotted time.

### 2.4 Statistical analysis

All data analyses were conducted using R (version 4.3.0; R Foundation for Statistical Computing, Vienna, Austria). Linear mixed-effects models (LMMs) were fitted to examine the effects of Gender and Experimental Condition on TES, including follow-up simple effects analyses and effect size calculations and Tukey’s method was applied for multiple comparison correction. A two-way between-subjects ANOVA was also performed with factors Gender and Trial Frequency, and Type III sums of squares were used to account for unequal group sizes (via the afex package). Statistical significance was set at *p* < 0.05, Additionally, regression analyses were performed with coefficient estimation, and sensitivity analyses were conducted to evaluate the robustness of the results. Intraclass correlation coefficients were calculated to assess within-group reliability.

G*Power 3.1 software was used for sample size estimation and power analysis.

For data visualization, we used Python (version 3.10.9. Python Software Foundation, Wilmington, DE, United States) to generate histograms, violin plots, and line charts, scatter plots, regression curves.

## 3 Results

**FIGURE 2:**
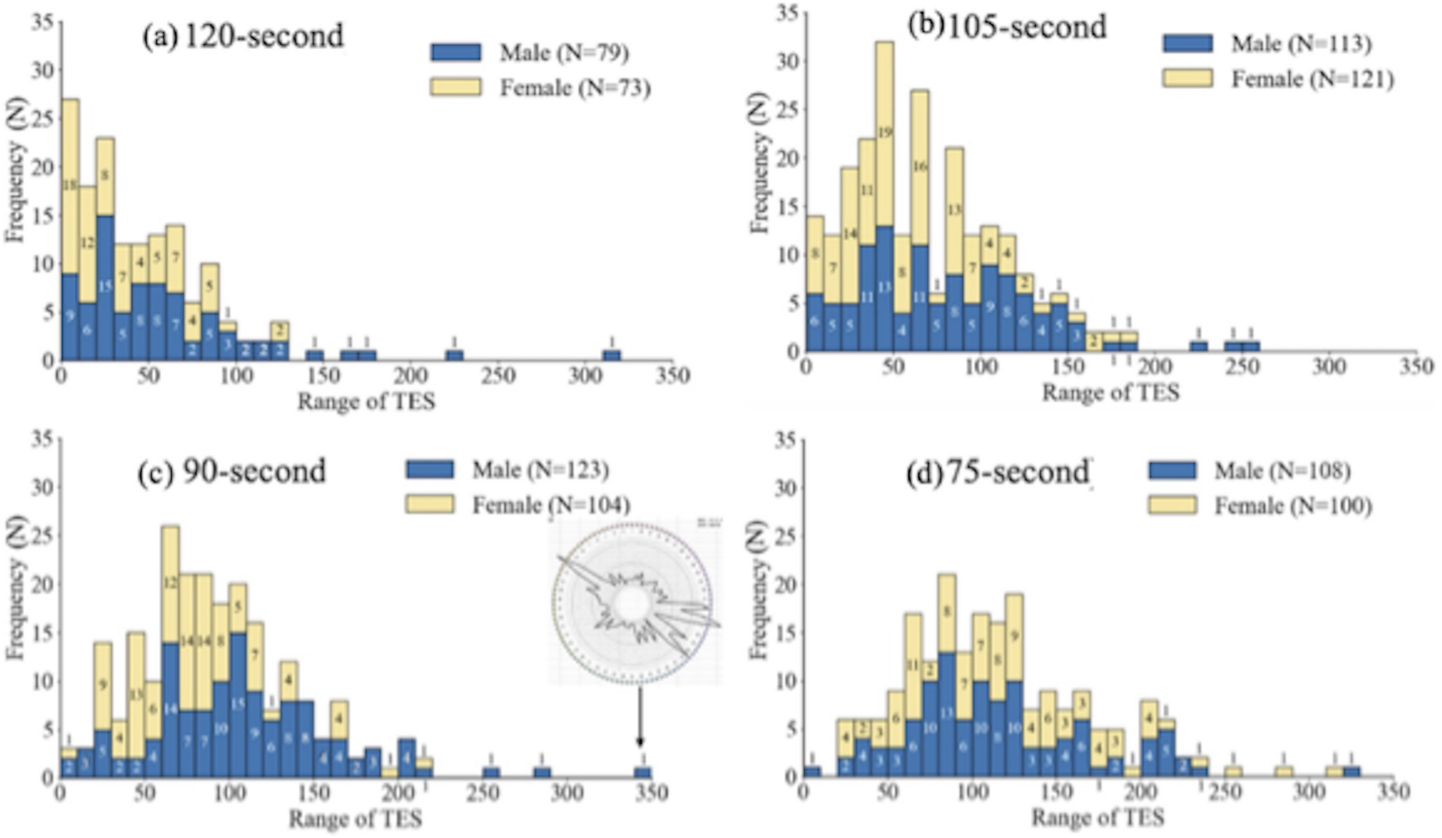
Distribution of TES (**a:** 120-s condition, **b:** 105-s condition, **c:** 90-s condition, and **d:** 75-s condition).

Figure 2 presents the distribution of TESs for males and females across the four time conditions: (a) 120, (b) 105, (c) 90, and (d) 75 s. he x-axes represent TESs in increments of 10, while the y-axes show stacked frequencies of. In each panel, the x-axes represent TESs in increments of 10, while the y-axes show stacked frequencies of participants within each TES range. Male participants are shown in blue (lower bars), and female participants are shown in yellow (upper bars). The total number of trials (N) for each condition is shown in the upper right corner of each corresponding plot. The numbers displayed in the middle of each bar indicate the number of participants within that TES range. A previous study (Kawabata et al., 2020) indicated that TESs above 150 in the 120 s condition and above 200 in the 90 s condition may suggest color vision deficiency. However, no reference criteria exist for 105 and 75 s conditions. Therefore, we examined the distribution patterns of participants with TESs exceeding 150 in the 120 and 105 s conditions, and those exceeding 200 in the 90 and 75 s conditions. Based on this evaluation, we identified one anomalous trichromat in the 90 s condition.

Participants completed the color discrimination task under one or more temporal conditions. The dataset was segmented by condition to enable independent evaluation of temporal effects, minimize carryover influences, and reduce inflation of TES values due to task unfamiliarity.

Following Kawabata et al. (2020), TESs above 150 in the 120 s condition and above 200 in the 90 s condition may indicate atypical color vision. One participant in the 90 s condition exceeded this threshold and was identified as a potential anomalous trichromat. This participant’s data was excluded from all subsequent analyses to ensure that the results reflected typical trichromatic performance. The individual’s TES distribution remains shown in the result of 90 s for transparency.

To facilitate verification of the main data across conditions, Table 3 summarizes the mean ± SD of TESs for male and female participants under each exposure-time condition. This table complements Figure 2 by providing precise numerical values for comparison between conditions and sexes. The experimental data presented in Figure 2 reveal a wide distribution of TESs among normal trichromats. Substantial individual variation was observed among participants around 20 years of age, who typically exhibit strong color discrimination abilities (Kinnear and Sahraie, 2002). Under the 120 s condition (Figure 2a), the peak TES range for males was 20–30, compared with 0–10 for females, and only males achieved TESs above 130. In the 0–20 and 30–40 ranges; however, females outnumbered males. Within the peak range, 24.66% of females and 18.99% of male were represented. In the 105- s condition (Figure 2b), both sexes peaked at 40–50, although only males scored above 190. In this peak range, 15.7% of females were represented compared with 11.5% of males, while females also predominated in the 0–30, 40–70, and 80–100 ranges. At 90 s (Figure 2c), one anomalous trichromat was identified, the participant’s pattern graph is shown as an inset, within an arrow indicating the corresponding TES range. The male peak was lower, at 70–90. Only males scored above 220, yet within the peak range, 26.92% of female and 12.20% of males were represented. Females exceeding males in seven intervals between 20 and 90. In contrast, the 75- s condition (Figure 2d) showed a different trend: the male and female peaks occurred at 80–90 and 60–70, respectively. Unlike longer time limits, TES values above 250 were recorded for both sexes, with females slightly more represented. In the peak range, 11% of females and 12.04% of males were observed. Notably, under this very short time limit, females exhibited a broader TES distribution, indicating reduced color discrimination compared with longer conditions.

**TABLE 3:** Mean and standard deviation (SD) of TESs for males and females across four exposure-time conditions.

Time limits	All participants					
	Total trials	Average(± SD)	Males (Trails)	Average(± SD)	Females (Trails)	Average(± SD)
120s	152	46.572(± 44.2045)	79	56.291(± 52.1244)	73	36.055(± 30.6412)
105s	234	69.043(± 46.3635)	112	79.327(± 51.0011)	122	59.438(± 39.4134)
90s	227	92.907(± 50.7861)	123	106.618(± 50.7861)	104	76.692(± 38.9830)
75s	208	114.442(± 56.2901)	108	114.111(± 55.5158)	100	114.800(± 57.3925)
**Time limits**	**Normal trichromats**					
	**Total trials**	**Average(± SD)**	**Males (Trails)**	**Average(± SD)**	**Females (Trails)**	**Average(± SD)**
120s	152	46.572(± 44.2045)	79	56.291(± 52.1244)	73	36.055(± 30.6412)
105s	234	69.043(± 46.3635)	112	79.327(± 51.0011)	122	59.438(± 39.4134)
90s	226	91.779(± 47.9604)	122	104.639(± 51.1981)	104	76.692(± 38.9830)
75s	208	114.442(± 56.2901)	108	114.111(± 55.5158)	100	114.800(± 57.3925)

Overall, the findings indicate that peak TES ranges across the different time limits were generally lower for females than for males, while the proportions within these ranges were higher among females. In several lower TES intervals, both the number and proportion of female participants exceeded those of males, suggesting that female TESs tended to be lower. This pattern supports hypothesis 1.

### 3.1 Gender

For further analysis, we excluded anomalous trichromats identified in the 90 s condition, leaving data from 355 normal trichromats.

A LMM was fitted with TES as the dependent variable, Gender and Condition as fixed effects, and Participant as a random intercept. The model formula was TES = Gender * Condition + (1/Participant), with random intercepts accounting for repeated measures within participants. The model successfully converged (Restricted Maximum Likelihood criterion = 8426), and residual inspection for extreme values (scaled residuals ranging from −3.42 to 4.45), indicating reasonable model fit.

Examination of fixed-effect correlations in the ordinary (non-centered) LMM showed a substantial correlation between the intercept and Condition (*r* = −0.949), suggesting multicollinearity that could obscure interpretation of main effects. To address this, the Condition variable was mean-centered before refitting the model. Variance inflation factors in the centered model were low (Gender: 1.00, Condition_c: 2.13, Gender × Condition_c: 2.13), confirming that multicollinearity was effectively reduced. Random effects variance components indicated that individual differences accounted for a variance of 1516 (SD = 38.9) for Participant intercepts, whereas residual variance was 890 (SD = 29.8), confirming that the model appropriately captured between-participant variability.

After centering Condition, the LMM revealed a significant main effect of Gender, with males showing higher TESs than females [β = 18.981, SE = 4.698, *t* (351.25) = 4.04, *p* < 0.001]. Effect sizes, expressed as partial η^2^ from a Type III ANOVA of the centered LMM, were 0.0526 for Gender, indicating a small effect according to conventional guidelines. These findings show that females generally exhibited lower TESs than males, consistent with superior color discrimination ability, supporting Hypothesis 1.

Although the main effect of Gender was statistically significant, the effect size was relatively small (0.0526), indicating a modest difference in TESs between males and females. This suggests that although females tended to show slightly better color discrimination, the practical impact of this difference is limited. Accordingly, the statistical significance should be interpreted with caution, and real-world implications of gender differences should consider the small effect size.

### 3.2 Experimental conditions

**FIGURE 3:**
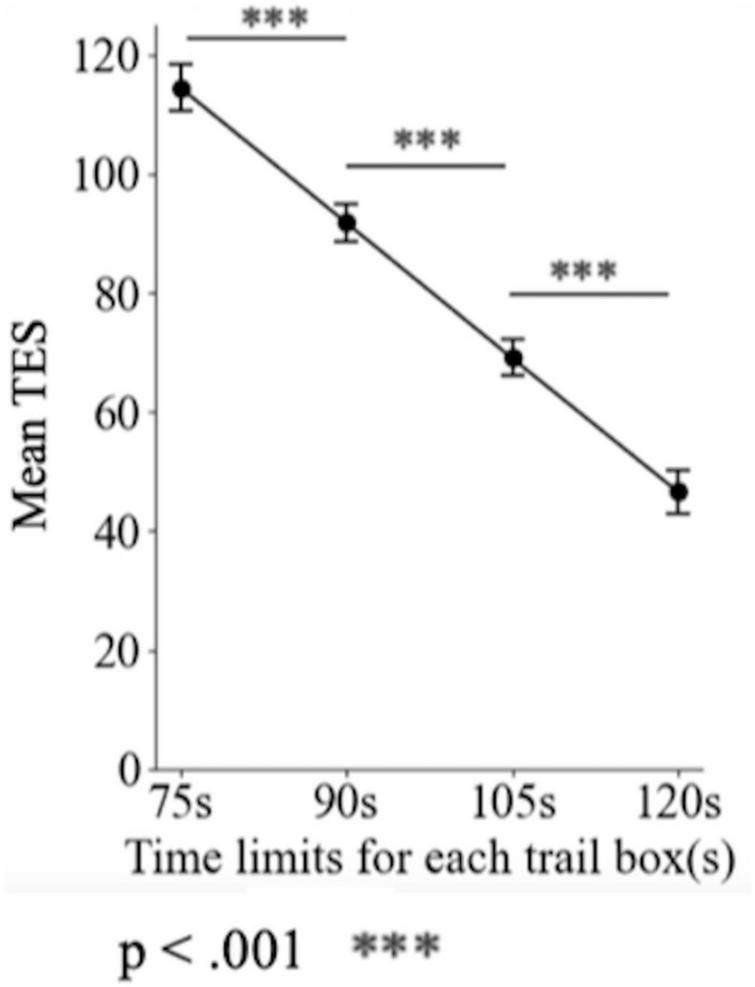
Variation in mean TES score under different time limit conditions. Error bars represent standard errors.

The LMM revealed a significant main effect of experimental conditions [β = −1.473, SE = 0.105, *t* (536.09) = −14.06, *p* < 0.001], indicating that TESs varied across conditions. Multiple comparisons adjusted using the Tukey Honest Significant Difference method showed significant differences among all four conditions, with all pairwise *p*-values below 0.0001. Figure 3 illustrates changes in mean TESs under different time limits, The x-axis represents the time-limit conditions, and the y-axis shows the mean TESs. Each point corresponds to the mean TES for that condition, with error bars indicating the standard error (SE) across participants. A clear downward trend emerged as the time limit increased: the mean TES decreased from 114.442 (± 56.2901) at the 75 s condition to 46.572 (± 44.2045) at 120 s condition. These findings suggest that shorter time limits impaired performance, thereby supporting hypothesis 2. The partial eta squared for the experimental condition was 0.5873, reflecting a large effect size on color discrimination ability.

### 3.3 Interaction between gender and experimental conditions

The analysis revealed a significant interaction between gender and experimental conditions [β = 0.415, SE = 0.144, *t* (525.09) = 2.88, *p* = 0.0041], indicating that the effect of time constraints on color discrimination performance varied by gender. A simple effects analysis by gender showed that females and males exhibited significant differences in TESs across all four experimental conditions Conversely, the simple effects analysis by condition revealed significant gender differences in TESs under the 120 s (*p* < 0.0001), 105 s (*p* < 0.0001), and 90 s (*p* < 0.0001) conditions, but no notable difference was observed under the 75 s condition (*p* = 0.4420). The effect size for the interaction between gender and condition was small (0.0204), indicating a minimal influence on color discrimination performance. To further investigate the significant Gender × Condition interaction in the LMM, simple effects analyses were performed.

Condition comparisons within each gender showed that TESs significantly decreased as the exposure time increased for females and males (Tukey-adjusted, all *p* < 0.05). For females, TES differences ranged from 13.9 to 67.9 across condition pairs, with the largest difference observed between 75 and 120 s (estimate = 67.9, SE = 5.15, *p* < 0.0001). For males, TES differences ranged from 14.1 to 47.1, with the largest difference between 75 and 120 s (estimate = 47.1, SE = 4.75, *p* < 0.0001). This pattern indicates that shorter exposure times impaired performance in both genders, with females showing larger changes across conditions.

Gender comparisons within each condition showed no significant difference at 75 s (estimate = −4.7, SE = 6.11, *p* = 0.442). However, females had significantly lower TESs than males at 90 s (estimate = −24.4, SE = 5.97, *p* < 0.0001), 105 s (estimate = −22.2, SE = 5.89, *p* < 0.0001), and 120 s (estimate = −25.5, SE = 6.80, *p* < 0.0001). These results indicate that female participants consistently outperformed male participants under prolonged exposure times.

**FIGURE 4:**
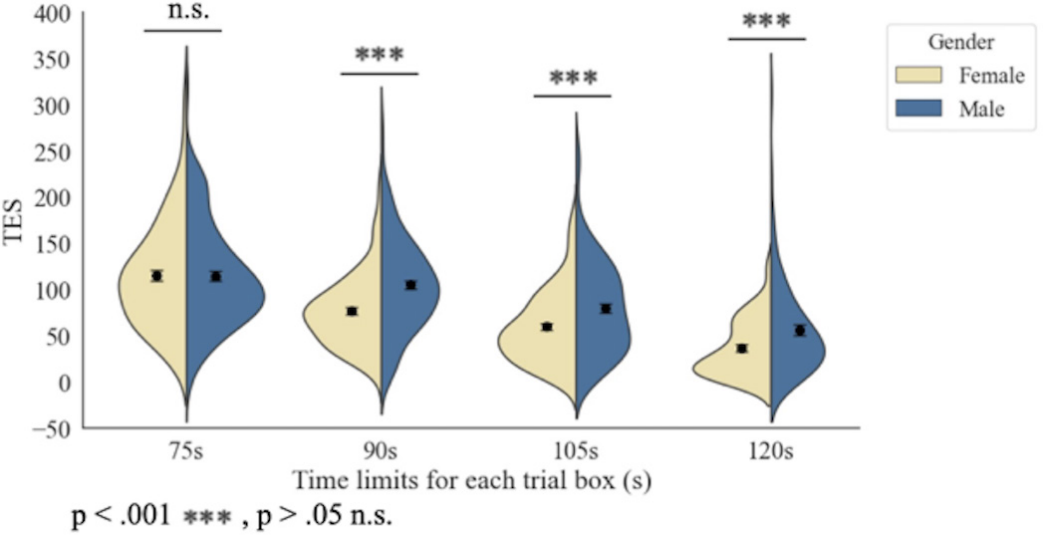
Distribution of TESs across time-limit conditions for male and female participants. Error bars represent standard errors.

Figure 4 shows TES distributions across the four time-limit conditions (75, 90, 105, and 120 s) for male and female participants. Colored violins represent the full TES distribution for each condition and gender (yellow: females, blue: males), with each violin split to display both distributions side by side. Black dots indicate the mean TES for each group and condition, and vertical error bars representing the SE across participants. The x-axis presents the experimental time-limit conditions, and the y-axis shows TES. Statistical significance between groups is marked with asterisks (***: *p* < 0.001; n.s.: not significant). The figure illustrates that females outperformed males in color discrimination under prolonged time limits (120, 105, and 90 s), whereas no performance difference was observed under the 75-s condition which is not consistent with H1.

As the linear LMM revealed significant Gender × Condition interaction, we further examined potential non-linear trends in TES across conditions by adding a quadratic term for the mean-centered Condition variable (Condition_c^2^) to the model: TES = Gender * Condition_c + Gender * Condition_c^2^ + (1/Participant). The model showed a significant positive quadratic effect of Condition_c^2^ [β = 0.02057, SE = 0.00728, *t*(520.13) = 2.82, *p* = 0.0049], indicating a slight curvature in TES across exposure times. The Gender × Condition_c^2^ interaction was marginally significant [β = −0.01740, SE = 0.01014, *t*(519.43) = −1.72, *p* = 0.0867], suggesting that the curvature differed slightly between males and females, with females showing a somewhat steeper change in TES across conditions.

Robustness check: To ensure that the LMM results were not unduly influenced by extreme values, a robust linear mixed-effects model (rlmer) was fitted using the same fixed and random effects structure. The robust model produced results consistent with the conventional LMM: males had higher TESs than females (β = 18.89, SE = 4.38, *t* = 4.32); Condition negatively affected TES (β = −1.31, SE = 0.09, *t* = −14.44); and the Gender × Condition interaction was marginally significant (β = 0.24, SE = 0.12, *t* = 1.92). Examination of the robustness weights showed that most residuals (721/820) and random effects (301/355) had weights close to 1, indicating that only a small subset of data points was downweighed. These findings confirm that the observed effects are robust to potential outliers.

### 3.4 SAT speed–accuracy tradeoff

The experimental results showed that participants’ color discrimination performance declined as the time limit decreased, demonstrating a SAT in both genders. Non-parametric bootstrap regression analyses were performed on individual participants’ TES scores to quantify this effect.

For females, a quadratic regression of TES on Condition yielded coefficients (95% bootstrap percentile CI) of intercept = 385.53 (216–567), linear term = −4.85 (−8.52 to −1.42), and quadratic term = 0.016 (−0.001 to 0.035), indicating a significant non-linear increase in ESs as time limits shortened. For males, an inverse regression of TES on Condition yielded coefficients of intercept = −28.94 (−61.99 to 2.38) and 1/Condition = 11250.59 (8319–14390), showing a similar increase in errors. Under shorter time limits, females’ TES increased more sharply than males’, particularly between the 90 and 75 s conditions, suggesting that extreme time constraints influenced female participants more strongly. These bootstrap results confirm that the observed SAT is robust and not disproportionately influenced by individual outliers or unequal trial repetitions.

**FIGURE 5:**
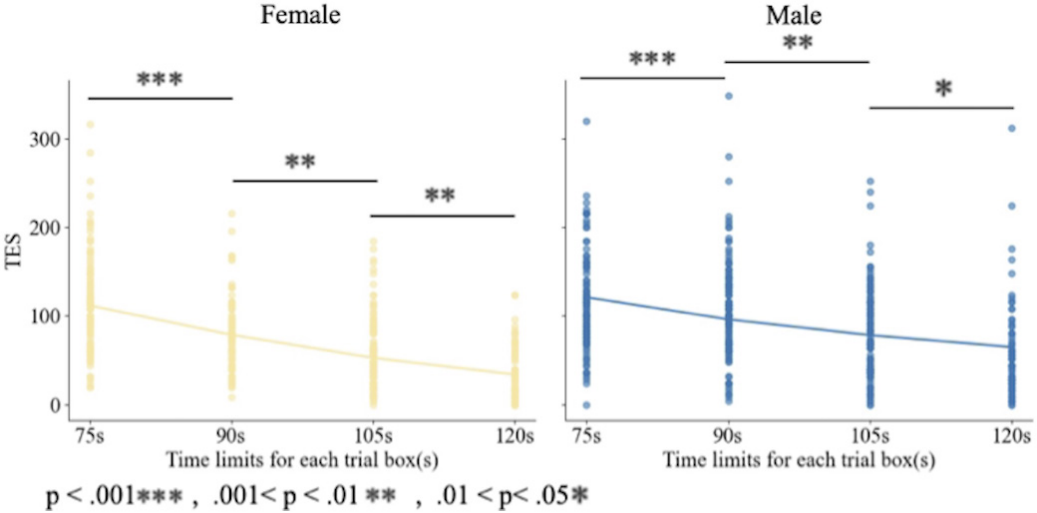
Results of SAT curves (the regression curves for females (left) and males (right) under four conditions).

Figure 5 presents scatter plots and fitted regression curves of TESs across the four time-limit conditions for all participants. The figure is divided into two panels: the left panel shows females (yellow), and the right panel shows males (blue). The x-axis represents the time-limit conditions for each trial, and the y-axis represents TES. Statistical significance is indicated with asterisks: *** for *p* < 0.001, ** for 0.001 < *p* < 0.01, and * for 0.01 < *p* < 0.05. The plots show that TES increases as the time limit decrease for both genders, although the rate of increase differs between females and males.

#### 3.4.1 Sensitivity analysis of regression models

To verify that the SAT patterns were not model-dependent, alternative regression models were fitted for each sex. For females, linear (TES = 237.49–1.702⋅Condition) and inverse (TES = −88.06 + 15137.47/Condition) models were evaluated alongside the original quadratic model. For males, linear (TES = 218.91–1.327⋅Condition) and quadratic (TES = 53.70 + 2.18⋅Condition – 0.0181⋅Condition^2^) models were evaluated alongside the inverse model. Across all models, TES consistently increased as time decreased, and the sex differences in the SAT trend remained evident. These results indicate that the reported SAT and gender effects are robust to the choice of regression model.

### 3.5 Number of times

To examine the effect of trial number on color discrimination performance in males and females, we conducted a two-way between-participants ANOVA with gender and number of trials as factors. The analysis revealed a significant main effect of trial number [*F*(3, 813) = 22.380, *p* < 0.001], while the interaction effect was negligible [*F*(3, 813) = 0.290, *p* = 0.836]. Under the 120 s condition, performance after four trials was considerably higher than in the first trial [*F*(1, 148) = 12.180, *p* < 0.001].

Similarly, in the 105 s condition, the first trial showed considerably lower performance compared to the second (*p* < 0.0001), third (*p* < 0.0001), and fourth trials (*p* = 0.0154). For the 90 s condition, the third trial yielded higher discrimination accuracy than the first (*p* < 0.0001) and second trials (*p* = 0.0022). In the 75 s condition, the fourth trial outperformed the first (*p* = 0.0192), second (*p* = 0.0218), and third trials (*p* = 0.0065).

These results indicate a consistent improvement in discrimination accuracy with increasing trial number. To mitigate potential inflation of ESs due to participants’ unfamiliarity with the procedure or equipment, we excluded first trial results. Although the number of participants varied across trial repetitions within each condition, weighted means were used to ensure consistency. After this adjustment, data across different time-limit conditions were comparable, all demonstrating the trend that color discrimination ability improves with repeated trials.

**FIGURE 6:**
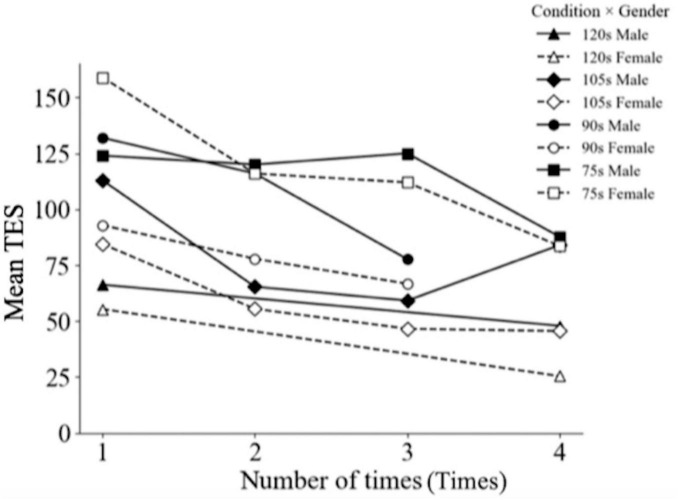
Variation in mean TES as a function of the number of trials for male and female participants.

Figure 6 shows changes in mean TESs for male and female participants across four trial repetitions. The x-axis represents the trial number (1–4), and the y-axis shows the mean TES. Different lines correspond to participant groups defined by gender and time-limit conditions, as indicated in the legend at the top right. The figure illustrates the changes in the mean TES as the number of test trials increases for both genders across all time-limit conditions. The data clearly show that error scores are highest during the first trial and progressively decrease with subsequent trials.

## 4 Discussion

### 4.1 Mechanisms underlying gender differences in the 120-, 105-, and 90-s conditions

The experimental results under the 120-, 105-, and 90-s conditions revealed a marked superiority in color discrimination among females compared with males (Figure 5), partly supporting our initial H1. This trend suggested that gender differences in visual performance emerge substantially under time pressure, raising important questions about the mechanisms underlying such differences.

Several explanatory frameworks may help interpret the observed gender differences in color discrimination. From an evolutionary psychology perspective, the superior performance of females may reflect adaptive advantages associated with foraging activities that required precise detection of spatial and chromatic cues (Silverman and Eals, 1992). Genetically, females possess two X chromosomes, which may encode distinct variants of red and green photopigments. Although rare, some evidence suggests that the simultaneous expression of these variants could broaden red–green sensitivity, a phenomenon sometimes referred to as “female tetrachromacy” (Panchal et al., 2012). While speculative and likely applicable to only a small subset of women, this may partially explain subtle female advantages in color discrimination observed in behavioral research. Neurobiological factors may also contribute. Females have been reported to exhibit increased interhemispheric connectivity (Mcgivern et al., 2019) and higher P-cell density (Hasegawa and Hayasaka, 2012), potentially supporting finer chromatic and spatial processing. Tomasi and Volkow (2012) further reported that females have ∼14% higher local functional connectivity density and ∼5% greater gray matter density than males in several cortical and subcortical regions, suggesting a neurobiological basis for more refined perceptual functions. These structural differences may underlie observed behavioral differences between males and females. Overall, biological factors combined with experience-dependent plasticity likely contribute to the observed gender differences. However, the present findings are behavioral and do not directly test biological mechanisms. Instead, the improvements observed across trials for both genders indicate that experience-dependent processes—such as perceptual learning and memory reinforcement—play a central role in shaping discrimination performance. This interpretation aligns with prior work showing that repeated engagement in color-sensitive tasks can improve perceptual sensitivity (Kasai and Kawabata, 2021; Sasaki and Kawabata, 2023). Therefore, while biological factors may account for baseline gender differences, the current results primarily support the view that perceptual plasticity dynamically modulates color discrimination under time constraints.

In general, the findings of this study suggest that the observed female advantage in color discrimination under time constraints may arises from the interplay of stable biological predispositions (e.g., genetics, neuroanatomy, and cell-type distribution) and dynamic experiential influences (e.g., repeated low-intensity perceptual practice). Placing these results within evolutionary, genetic, and neurobiological frameworks underscores that gender differences in visual performance reflect not a single cause but the integration of predispositions and plasticity. Therefore, this research reinforces the mechanistic understanding of color perception by demonstrating how biological and experiential factors jointly influence observed performance differences.

### 4.2 Mechanisms underlying the absence of gender differences under the 75-s condition

Our results revealed that males and females performed comparably in color discrimination under the shortest time-limit constraint (75 s), which is not consistent with H1. This result suggests that extreme time pressure alters the mechanisms that normally produce gender-related differences.

One mechanism is that severe time constraints restrict the deployment of analytical and experience-based strategies. In our data, the female advantage increased with longer time limits (90–120 s), consistent with prior findings that females benefit from accumulated everyday experience in color-related tasks (Kawabata et al., 2020). Meanwhile, the 75-s condition eliminated this advantage, suggesting that participants were forced to rely on rapid, intuitive judgments that diminish the experiential benefits typically favoring females.

A second mechanism is that males’ biological motor advantage compensates under extreme constraints. Lipps et al. (2013) reported that males show shorter motor execution times and faster wrist acceleration. Our data align with this; although female accuracy dropped sharply from 0.81 (120 s) to 0.71 (75 s), male accuracy showed less decline (from 0.74 to 0.70). This relative stability suggested that faster motor execution enables males to sustain their performance under high time pressure, thereby reducing the accuracy gap with females. Moreover, structural and functional brain differences may contribute to this pattern. Ingalhalikar et al. (2014) reported that male brains tend to favor connections that support perception-to-action integration, whereas female brains facilitate communication between analytical and intuitive networks. Under extreme time constraints, increased reliance on rapid sensorimotor pathways—which are more advantageous for males—likely reduces the contribution of experience- and analysis-dependent strategies that typically benefit females. This provides a neurophysiological explanation for the convergence of male and female performance under the 75-s condition. Overall, the findings indicate that the absence of gender differences under the 75-s condition is mechanistically driven rather than incidental. Extreme time pressure suppresses experience-based and detail-oriented processing, which normally favors females, while simultaneously amplifying the role of motor execution speed, which favors males. By placing this pattern within the theoretical frameworks of decision thresholds and adaptive strategy selection, our results suggest that cognitive and motor systems dynamically adjust to environmental constraints, thereby explaining why gender differences emerge under moderate time limits but do not under extreme temporal constraints.

### 4.3 Mechanisms underlying the SAT in color discrimination

Our results revealed clear differences in performance across the four time conditions (120, 105, 90, and 75 s). Peak accuracy was observed under the 120-s condition, whereas the lowest accuracy was observed under the 75-s condition (Figure 3). The TES also increased systematically as the available time decreased (see Figure 3), demonstrating a robust SAT. These findings partly support our H2 that color discrimination performance across the full hue circle is expected to decrease progressively as time limits are shortened.

This pattern suggests a mechanism in which time pressure limits the engagement of analytical and experience-based strategies. Under the 120-s condition, participants could carefully observe, sort, and adjust the colored caps, leading to higher accuracy. In contrast, under the 90- and 75-s conditions, participants increasingly relied on rapid, intuition-driven judgments, resulting in more errors. The trends shown in Figure 3 provide empirical support for this mechanism, illustrating that decreased time limit systematically reduces accuracy while increasing TES.

These results align with prior findings. Neurophysiological evidence (Yau et al., 2020) suggests that caudate nucleus activity modulates the SAT: higher activity slows decisions and enhances accuracy, whereas lower activity reflects disinhibition, leading to faster but less accurate responses. This is consistent with our findings that under extreme time pressure, participants relied more on rapid, intuition-based judgments. Kawabata et al. (2020) reported lower TESs under longer observation times, and Yellott (1971) demonstrated that the red–green discrimination accuracy fluctuates with time constraints. Gender-related evidence from Mcgivern et al. (2019) further indicates that although females generally achieve higher accuracy, they require longer response times, reflecting a prioritization of accuracy over speed. Stimulus properties also play a role: the ND-100 hue test performed in this study involved colored caps with low luminance and subtle chromatic differences (average ∼ 0.9 NBS units), which amplified the effects of shortened viewing time on accuracy (Figure 3).

Mechanistically, these findings suggest that under high time pressure, participants shift from careful, detail-oriented, and experience-driven processing toward rapid, intuition-based processing. This shift is modulated by task demands, stimulus difficulty, and individual decision thresholds. Our results, in conjunction with theoretical SAT models (Heitz, 2014), indicate that cognitive systems dynamically adjust attention and decision thresholds in response to environmental constraints.

In summary, the SAT in color discrimination arises from the interplay between time-constraint-based suppression of analytical and experience-based strategies and increased reliance on fast, intuitive responses. The patterns observed in Figure 3 provide direct evidence for this mechanism, placing the phenomenon within established theoretical frameworks.

### 4.4 Mechanisms underlying gender differences in the SAT

Although males and females showed significant differences in discrimination performance across the four time conditions, the increase in TES from 120 to 75 s was greater for females than for males, resulting in no significant TES difference between genders under the 75-s condition. This finding contradicts H2, indicating that gender differences in the SAT emerge specifically under high time pressure, highlighting potential underlying mechanisms.

One mechanism relates to decision-making strategies and motor initiation. Heitz and Schall (2012) proposed that SAT regulation occurs primarily during decision formation and motor preparation. Females tend to prioritize accuracy by extending response time, but under the fixed 75-s constraint, this strategy becomes infeasible, preventing compensation for reduced processing time and resulting in lower accuracy. In contrast, males may adopt a more consistent, speed-stable strategy, yielding minimal performance variation across these conditions. Our data support this; the mean TES for females dropped by 38.108 when the time limit was reduced from 90 to 75 s, whereas that for males remained nearly constant (only dropped by 9.472) (Figure 4).

The second mechanism involves neural resource recruitment. Functional magnetic resonance imaging (fMRI) studies indicate that under high cognitive load, females show widespread activation in regions such as the dorsolateral prefrontal cortex, premotor area, supplementary motor area, and frontal eye fields (Jiang et al., 2024), reflecting engagement in rule switching and motor preparation. Males, however, rely primarily on the frontal eye fields and supplementary motor area, emphasizing attention orientation and automated motor sequences. These neural activation patterns align with our behavioral data; the greater performance decline in females under the 75-s condition suggested that time constraints prevented the full utilization of these resource-intensive strategies.

Third, differences in decision-threshold adjustment contribute to gender disparities. Forstmann et al. (2011) reported that higher thresholds favor slower, more accurate responses, while lower thresholds facilitate faster, potentially less accurate responses. Under the 75-s condition, females may have been forced to lower their thresholds to meet time demands, sacrificing accuracy, whereas males maintained stable thresholds, sustaining performance. Individual differences in threshold adjustments have been shown to correlate selectively with activation in the striatum and pre-supplementary motor area (Forstmann et al., 2008), suggesting a neural basis for the observed behavioral patterns. This was also reflected in the behavioral data; when the time limit was reduced to 75 s, the TES of females decreased more than that of males (Figure 4).

Collectively, these findings suggest that gender differences in the SAT arise from the interaction of cognitive strategies, neural activation patterns, and decision-threshold adjustments under high time pressures. Females’ accuracy-focused strategies are constrained under strict time limits, whereas males’ more automated and stable processing enables the achievement of consistent performance. The consistency of our experimental data with prior theoretical models (Heitz and Schall, 2012; Forstmann et al. (2011); Jiang et al., 2024) reinforces the mechanistic understanding: gender-specific outcomes in the SAT result from the dynamic interplay of neurocognitive resources, strategy selection, and task-imposed temporal constraints.

### 4.5 Explaining the effect of the number of trials on discrimination ability

Our analysis revealed a clear pattern in which the discrimination performance improved across successive trials, indicating a learning or adaptation effect (Figure 6). Notably, there was no statistically significant interaction between gender and trial number, suggesting that the improvement was consistent across males and females. This phenomenon indicates that repeated exposure facilitates perceptual learning independent of the gender.

Mechanistically, the results suggest that practice or repeated engagement in the task allows participants to refine perceptual strategies and optimize decision thresholds Neurophysiological evidence supports this mechanism: after training, V1 subregions corresponding to the trained visual field quadrant show increased activation alongside improved task performance (Yotsumoto et al., 2008). As trials progress, participants become more familiar with the color caps and task demands, enhancing discrimination accuracy and stabilizing response patterns. The ANOVAs and *post hoc* comparisons within each time-constraint condition confirmed this trend; accuracy increased from trial 1 to trial 4 across all four time conditions (Figure 6), providing direct data support for the role of experience-dependent perceptual adaptation.

This learning effect aligns with existing theoretical frameworks on perceptual plasticity and experience-dependent learning (Kasai and Kawabata, 2021; Sasaki and Kawabata, 2023). Repeated exposure strengthens the processing of subtle chromatic differences and stabilizes attentional allocation, allowing participants to optimize performance regardless of inherent gender differences. The controlled sequence of trials across time-constraint conditions ensured approximately equal exposure (see the section 2.1), minimizing potential confounds and confirming that the observed gender differences in the SAT and discrimination performance cannot be attributed to the trial number.

In summary, the improvement in the discrimination performance across successive trials reflects an experience-dependent, trial-based learning mechanism. This mechanism operates consistently across genders and time constraints, providing evidence that perceptual adaptation enhances color discrimination independently of other experimental manipulations. The data shown in Figure 6 support this conclusion, placing the observed trial effects within established models of perceptual learning and cognitive plasticity.

### 4.6 Mechanistic interpretation of gender performance differences under time pressure

The present results indicate that gender differences in color discrimination emerge from the dynamic interplay of three mechanisms—biological predispositions, experience-dependent perceptual plasticity, and adaptive cognitive strategies—with time pressure acting as a key modulator.

Biological predispositions provide the foundational advantage for females under moderate time constraints (90–120 s). Female participants exhibited higher average accuracy than males (Figure 4) (“Mean TES), consistent with enhanced red–green photopigment variation (Panchal et al., 2012), greater P-cell density (Hasegawa and Hayasaka, 2012), and stronger interhemispheric connectivity (Mcgivern et al., 2019). These physiological factors supported fine chromatic discrimination and spatial processing, but their effect was constrained when time was extremely limited (75 s), illustrating how environmental pressure modulates the manifestation of innate advantages.

Experience-dependent perceptual plasticity further refined performance. Across trials 2–4, accuracy increased significantly, consistent with prior findings on perceptual learning (Kasai and Kawabata, 2021; Sasaki and Kawabata, 2023). This trial-based improvement highlights that repeated exposure enables participants to optimize perceptual strategies, independent of gender; however, its benefits are the most evident when sufficient time is available, which allows engagement with task-relevant cues, demonstrating the interaction between experience and temporal constraints.

Adaptive cognitive strategies and decision-threshold adjustments affect performance under extreme time pressures. Females demonstrated a larger TES decline when the time limit was reduced from 90 to 75 s (38.108) than males (9.472), indicating that time constraints hinder the implementation of accuracy-focused strategies, forcing reliance on rapid, intuitive judgments. These findings align with SAT models and decision-threshold regulation (Heitz and Schall, 2012; Forstmann et al., 2011). Males’ stability under similar conditions suggested that automated, less resource-intensive strategies confer resilience to high temporal pressures.

Together, the findings support a preliminary triple-mechanism model in which (i) biological predispositions (e.g., photopigment, P-cell density, and interhemispheric connectivity variations), (ii) experience-dependent perceptual plasticity (as reflected in trial-based improvements), and (iii) adaptive cognitive strategies (e.g., decision-threshold adjustments under high time pressures) interact dynamically. This framework provides a mechanistic understanding of how gender differences in color discrimination emerge and attenuate for varying temporal constraints. Crucially, time pressure serves as a modulating variable, altering the relative contribution of each mechanism. Under moderate time limits, biological and experiential factors dominate, eliciting an advantage for females. Under extreme time limits, cognitive-strategy shifts and motor execution attenuate gender performance differences. This integrative framework bridges behavioral evidence with theoretical accounts from visual cognition, perceptual plasticity, and SAT models, providing a comprehensive explanation for the observed gender-specific patterns.

### 4.7 Limitations and prospects

This study has several limitations that suggest important directions for future research. First, the participant sample was homogeneous, consisting of Asian university students aged 18–28 with similar educational backgrounds. This limitation may influence the generalizability of the findings to other age groups, cultural backgrounds, or individuals with different educational experiences. Cultural differences in color perception or experience may influence experimental outcomes (e.g., Bortolotti et al., 2023; Singh and Srivastava, 2011; Bortolotti et al., 2025). To address this limitation, future research should recruit a more diverse sample, including participants from different age groups, occupational backgrounds, and ethnicities. Additionally, stratified sampling or multisite studies could enhance external validity and provide a more comprehensive understanding of color discrimination performance across populations.

Second, this study examined overall gender differences in color discrimination without analyzing specific hues. Notably, females show higher RG CAD detection thresholds than males (Rodríguez-Carmona et al., 2008), indicating lower RG sensitivity. Future studies should systematically examine hue-specific gender differences by designing experiments that isolate individual color axes (e.g., RG, BY) and include sufficient trials for each hue to allow robust statistical comparisons. Although data from the second to fourth trials were included, variations in the number of trials across experimental conditions limited a systematic analysis of perceptual plasticity and practice effects. To overcome this limitation, future studies should employ refined experimental designs that ensure an equal number of trials per condition and participant, potentially using adaptive trial assignment or counterbalancing to control for practice effects. Such approaches would enable a more precise assessment of how repeated exposure and experience shape color discrimination performance over time.

Third, although neural activity was not directly measured in this study, the observed interaction effects provide indirect behavioral evidence that males and females differ in color discrimination under time pressures. Future studies should directly investigate the neural mechanisms underlying these gender-specific effects using neuroimaging techniques such as fMRI or EEG in combination with the ND-100 hue test. For example, event-related potentials could examine temporal dynamics of color processing, while fMRI could identify brain regions showing differential activation patterns between males and females under varying time constraints. Integrating behavioral measures with neural data in a multimodal approach would allow testing specific hypotheses about the neural substrates of SATs and perceptual plasticity, thereby providing a mechanistic explanation for the observed gender differences.

Fourth, participants were tested in small groups of four in the same room, with approximately equal numbers of males and females and careful seating arrangements to prevent friends from being in the same group. Although each participant had an individual table and standardized illumination, group testing may still introduce subtle social or competitive influences that could influence performance. Future studies could conduct individual testing to completely eliminate potential social effects and ensure that performance differences reflect only perceptual and cognitive factors.

## 5 Conclusion

This study is the first to systematically investigate the effect of gender differences on color discrimination under extremely short time limits, addressing a key gap in understanding how time pressure shapes perceptual performance. Our results reveal a clear pattern: females outperform males under moderate time constraints (90–120 s), whereas the performance is consistent for both under extreme time pressures (75 s), highlighting time pressure as a key modulator of gender performance differences.

Mechanistically, these findings reflect the dynamic interplay of three interacting factors: physiological predispositions, cognitive strategies, and experience-dependent perceptual plasticity. Physiologically, females exhibit genetic variations in photopigments, higher P-cell density, and greater interhemispheric connectivity than males, supporting fine color discrimination. Cognitively, time constraints force dynamic adjustments in decision thresholds and strategy selection: under short durations, participants shift from detailed, accuracy-focused processing to rapid, intuition-driven responses. Experience-dependent mechanisms further modulate the discrimination performance as repeated exposure and perceptual practice enhance sensitivity, particularly benefiting females under longer time conditions. Under extreme time pressures, the compensatory effect of males’ faster motor execution interacts with the suppression of females’ detailed and experience-based strategies, leading to consistency in the discrimination performance.

The theoretical significance of this study is twofold. First, it uncovers the dynamic nature of gender differences in color discrimination, filling a gap in the literature by demonstrating that these differences are not fixed but emerge from the interaction of biological predispositions, adaptive cognitive strategies, and experience-dependent perceptual plasticity, which are dynamically modulated by environmental constraints, such as time pressure. Second, this study provides preliminary empirical support for a triple-mechanism model, highlighting how physiological factors (e.g., photopigment variations, neural connectivity variations, and different cell-type distributions), cognitive strategies, and perceptual experience collectively contribute to the observed performance differences and SAT. Together, these findings offer a mechanistic framework for understanding gender-specific perceptual performance and lay the foundation for future research to empirically test, refine, and extend our model to offer deeper insights into the interplay among biological, cognitive, and experiential influences under varying environmental conditions.

The findings of this study have significant practical implications for visual cognition research and real-world applications. By elucidating how gender-specific cognitive strategies, decision thresholds, and perceptual plasticity interact under time pressures, these results provide guidance for task design and performance optimization in applied settings. In professions where rapid and accurate color discrimination is critical—such as medical diagnostics, aviation, transportation, emergency response, quality control, and industrial operations—understanding these mechanisms can help minimize errors, improve response speed, and enhance decision accuracy.

The demonstrated role of experience-dependent perceptual plasticity suggests that structured training programs can effectively enhance color discrimination performance and stability under challenging conditions. In addition, insights into gender-specific differences in cognitive adaptation can inform the design of human–machine interfaces, safety protocols, and task workflows, ensuring that systems are tailored to optimize performance for diverse users. More broadly, the findings offer a framework for developing interventions, training regimens, and interface designs that leverage biological predispositions and experience-driven improvements, ultimately increasing efficiency, safety, and reliability in real-world color-dependent tasks.

## Data Availability

The datasets presented in this study can be found in online repositories. The names of the repository/repositories and accession number(s) can be found in the article/supplementary material.
